# Classification and mutation prediction based on histopathology H&E images in liver cancer using deep learning

**DOI:** 10.1038/s41698-020-0120-3

**Published:** 2020-06-08

**Authors:** Mingyu Chen, Bin Zhang, Win Topatana, Jiasheng Cao, Hepan Zhu, Sarun Juengpanich, Qijiang Mao, Hong Yu, Xiujun Cai

**Affiliations:** 1 Department of General Surgery, Sir Run-Run Shaw Hospital, Zhejiang University, 310016 Hangzhou, China; 2 Key Laboratory of Endoscopic Technique Research of Zhejiang Province, Sir Run-Run Shaw Hospital, Zhejiang University, 310016 Hangzhou, China; 3 Engineering Research Center of Cognitive Healthcare of Zhejiang Province, 310003 Hangzhou, China; 4 Zhejiang University School of Medicine, 310000 Hangzhou, China

**Keywords:** Cancer imaging, Hepatocellular carcinoma, Cancer models

## Abstract

Hepatocellular carcinoma (HCC) is the most common subtype of liver cancer, and assessing its histopathological grade requires visual inspection by an experienced pathologist. In this study, the histopathological H&E images from the Genomic Data Commons Databases were used to train a neural network (inception V3) for automatic classification. According to the evaluation of our model by the Matthews correlation coefficient, the performance level was close to the ability of a 5-year experience pathologist, with 96.0% accuracy for benign and malignant classification, and 89.6% accuracy for well, moderate, and poor tumor differentiation. Furthermore, the model was trained to predict the ten most common and prognostic mutated genes in HCC. We found that four of them, including *CTNNB1*, *FMN2*, *TP53*, and *ZFX4*, could be predicted from histopathology images, with external AUCs from 0.71 to 0.89. The findings demonstrated that convolutional neural networks could be used to assist pathologists in the classification and detection of gene mutation in liver cancer.

## Introduction

Hepatocellular carcinoma (HCC) is the fourth leading cause of cancer-related mortality and is currently the main cause of liver-related death, leading to more than one million deaths annually worldwide^1–3^. Over several decades, substantial progress had been made in the understanding of HCC risk factors, epidemiology, and molecular pathogenesis. The early detection of HCC increases the chance of curative therapies in high overall survival. Unfortunately, most HCC patients are diagnosed at the intermediate to late-stage, which significantly decreases the overall survival^4^. Various predominant clinical risk factors for the development of HCC have been defined, including alcohol abuse, cirrhosis, metabolic syndrome, and hepatitis B and/or C virus infection^5–8^. However, multiple genetic alternation and signaling cascades also have a great influence on tumor progression and overall survival^9^.

The understanding of HCC molecular pathogenesis has been significantly improved over the past decade^10^. The development of genomic analysis has identified the major drivers that are responsible for cancer development and progression. HCC has been reported to have around 40 genomic aberrations, some of which are deemed as drivers. Several frequent HCC genomic alternations have been identified, including mutations in the *CTNNB1* (β-catenin WNT pathway activation), *TP53*, telomere reverse transcriptase (telomere maintenance), AT-rich interaction domain 1A (*ARID1A*; chromatin remodeling), mammalian target of rapamycin signaling, *RAS* signaling, oxidative stress pathway activation, and aberrations in DNA methylation^11^. Previous studies have reported that the heterogeneity of HCC at both molecular and histological levels are correlated with gene mutations and oncogenic pathways^12^. The mutually exclusive *CTNNB1* (40%) and *TP53* (21%) mutations have been identified as two major groups of HCC according to its distinct phenotype. *CTNNB1* mutated HCC is generally well-differentiated and large, with pseudoglandular and microtrabecular patterns, and lacks inflammatory infiltrates; whereas *TP53* mutated HCC is generally poor-differentiated, with compact patterns, frequent vascular invasion, and pleomorphic, multinucleated cells^13^. The deeper understandings of the HCC phenotypes are essential for improving targeted therapies and clinical translation.

Pathologists could provide limited information regarding cancer reorganization from normal liver tissue and assess its histopathological grade via visual inspection, but it still lacks the underlying biological differences in HCC gene mutations associated with overall survival. The recent advances in artificial intelligence (AI) provided a novel way to assist clinicians to classify medical information and images^14–17^. Recently, Lin et al.^18^ used multiphoton microscopy with deep learning in the automated classification of HCC differentiation. Furthermore, Li et al.^19^ combined extreme learning machine with multiple convolutional neural network methods for nuclei grading in HCC. The development of graphics processing units allows the possibility to train a more complex neural network to satisfy the requirement of accomplishing complex visual recognition tasks, such as distinguishing tumors from normal tissue slides and classifying subtypes of tumors^20,21^. To the best of our knowledge, a previous study by Coudray et al.^20^ utilized the deep convolutional neural network on histopathological images to automatically classify the type and subtype of lung tumors. In addition, a promising result for the classification of colorectal^22,23^ and breast tumors^24^ using deep learning was also reported. Therefore, deep-learning models could be used to assist pathologists to effectively detect gene mutations and cancer subtypes. However, it remains unclear whether deep learning can be applied to solid tumors, especially for HCC. In addition, advances in AI tools in digital pathology have resulted in an increased demand for predictive assays in frozen slides that enable the selection and stratification of patients for additional treatment during surgery^25^.

Herein, based on the inception V3 network developed by Google^26^ and some packaging code from Coudray et al.^20^ via EASY DL platform and whole-slide images (WSIs) of H&E stained liver tissue, we have established a model to classify liver tissue and predict certain gene mutations. The model was externally validated by an independent cohort.

## Results

### The distribution of WSIs and tiles

There were 491 WSIs of H&E stained liver tissue from the Genomic Data Commons portal (GDC-portal, https://portal.gdc.cancer.gov/), including 402 WSIs of HCC and 89 WSIs of normal liver tissue. The information on histopathological grade was not available in 19 of 402 WSIs of HCC. According to the histopathological grade, they were then sorted into well (G1, *n* = 55), moderate (G2, *n* = 187), and poor group (G3/G4, *n* = 141) in the remaining 383 WSIs of HCC. A total of 387 WSIs of HCC with corresponding gene mutation information were available. Besides, 67 WSIs of HCC with histopathological grade and related gene mutation information and 34 WSIs of normal liver tissue were selected from Sir Run-Run Shaw Hospital (SRRSH). After each WSI was cropped into small “Tiles”, there are 119,596 “Tiles” (HCC vs. normal liver tissue, 87,422 vs. 32,174), 84,149 “Tiles” with histopathological grade (well vs. moderate vs. poor, 14,713 vs. 41,370 vs. 28,066) and 86,323 “Tiles” with corresponding gene mutation information. The distribution of WSIs and tiles was summarized in Table 1.

**Table 1:** The distribution of patients, histopathological images/WSIs, and tiles in each subset.

	Patients				Histopathological images				Tiles			
	Tr	Te	IV	EV	Tr	Te	IV	EV	Tr	Te	IV	EV
*Normal and HCC*												
HCC	208	41	128	67	225	47	130	67	41,578	8157	24,294	13,393
Normal	53	9	27	34	53	9	27	34	12,614	2204	9493	7863
*Histopathological grade*												
Well (G1)	31	7	14	17	33	8	14	17	6967	1893	2654	3199
Moderate (G2)	98	17	60	38	106	20	61	38	18,754	3862	10,953	7801
Poor (G3/G4)	69	14	48	12	76	16	49	12	13,701	3189	8783	2393
*CTNNB1* mutation												
Yes	60	13	26	21	63	15	26	21	11,283	3218	5329	4120
No	142	29	96	46	153	32	98	46	28,437	6321	18,342	9273
*FMN2* mutation												
Yes	31	7	9	10	32	7	9	10	6335	1632	2143	2736
No	171	35	113	57	184	40	115	57	34,103	7963	20,754	10,657
*TP53* mutation												
Yes	64	14	42	20	68	14	43	20	12,537	2873	7794	4341
No	138	28	80	47	148	33	81	47	26,521	6359	16,646	9052
*ZFX4* mutation												
Yes	35	5	20	15	36	6	20	15	7273	1468	3892	3224
No	167	37	102	52	180	41	104	52	33,219	7845	19,233	10,169

*Tr* training subset, *Te* test subset, *IV* internal validation subset, *EV* external validation subset.

### Deep learning framework

**Fig. 1:**
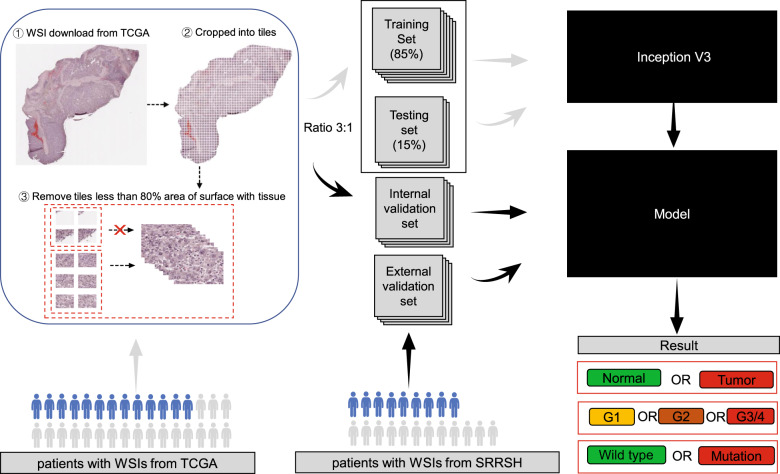
Deep-learning framework for training and evaluating the model to classify and predict mutation. Patients from TCGA were randomly divided into training cohorts (training and test) and internal validation cohort. Some patients had multiple virtual slides, and each slide was sliced into smaller “tiles”. The training, test, and internal and external validation sets were made up of multiple tiles from related cohorts. Model selection was done based on the performance in the test set. After learning and selection, the model was applied to tiles in the internal and validation sets to assess their performances.

Patients from GDC-portal were selected and identified as the primary cohort. Based on a random split-sample approach, a total of 377 patients were then randomly divided into a training cohort (consisting of testing cohort) and an internal validation cohort with a ratio of 3:1. In addition, 67 patients from our medical center were identified as an external validation cohort. All WSIs were cropped into multiple small “tiles” at a magnification of 20×. Finally, the training and testing set consisting of a large collection of tiles were used to train a neural network (inception V3) for the classification of liver tissue via EASY DL. The internal and external validation was performed by the remaining tiles from internal and external validation sets, respectively (Fig. 1).

### Performance of classification

**Fig. 2:**
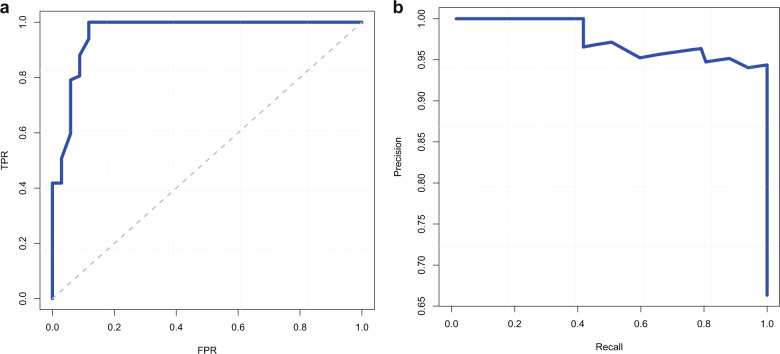
The performance of the model at automated recognizes tumors from normal liver tissue. **a** The receiver operating characteristic curve. TPR represents true positive rate, and FPR represents false positive rate. **b** Precision-recall curve.

The high-performance level of our models at recognizing tumors from normal liver tissue (AUC = 0.961; 95% CI 0.939–0.981) was observed in the validation set (Fig. 2a). Based on the class-imbalanced problem, the precision-recall curves (PR-curves) and Matthews correlation coefficient (MCC) were also used to evaluate its performance (Fig. 2b). The MCC was up to 0.82 for benign or malignant classification, and 0.738 for assessing histopathological grade (well, moderate, or poor). Compared to three pathologists with 2-year, 5-year, and 10-year experience in respective, the performances of our classifiers nearly reached the ability of pathologists with 5-year experience (Table 2).

**Table 2:** The performance of our models and pathologists’ ability for classification.

Classifiers	Performance	Our models	Pathologists with different years’ experience		
			2-year	5-year	10-year
Normal vs. tumor	Accuracy	0.960	0.911	0.970	0.990
	Precision	0.945	0.926	0.957	0.985
	Recall	1.000	0.940	1.000	1.000
	*F*1-score	0.971	0.933	0.978	0.993
	MCC	0.912	0.799	0.934	0.977
Well (G1) vs. moderate(G2) vs. poor (G3/G4)	Accuracy^a^	0.896	0.851	0.910	0.955
	Precision^a^	0.879	0.831	0.869	0.944
	Recall^a^	0.771	0.758	0.807	0.895
	Micro *F*1-score	0.820	0.754	0.836	0.914
	MCC^a^	0.738	0.637	0.764	0.882

*MCC* Matthews correlation coefficient.

^a^Average value.

### Performance of mutation prediction

**Fig. 3:**
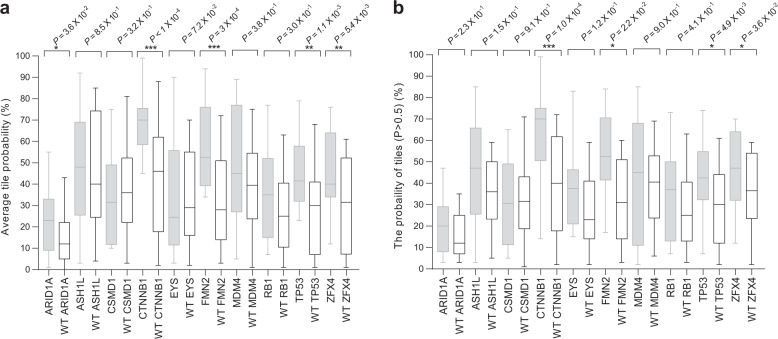
Prediction of the ten most common mutated genes in liver cancer using our deep-learning model and histopathology images. **a** comparison of the mutation and wild type in the distribution of the mutation probability in genes from tiles. **b** comparison of the mutation and wild type in the distribution of the mutation probability (Predicted *P* > 0.5) in each slide. *P* values were estimated with the two-tailed Mann–Whitney *U*-test (*P ≤ 0.05; **P ≤ 0.01; ***P ≤ 0.001). For the two box plots, the middle line within the box represents the median; box limits represent 95% upper and lower quartiles; and whiskers represent the minima and maxima.

Our models were trained and validated based on the ten most significantly mutated genes to estimate the possibility of mutation. The performances, including accuracy, precision, and recall rate, *F*1-score, and MCC, were summarized in Table 3. In order to reduce heterogeneity, the performance was assessed both the average predicted probability on region (tiles)-level and the probability of predicted tile (*P* > 0.5) on slide-level in the external validation set. On the region(tiles)-level, we found that five of which, including *ARID1A* (*P* = 0.036)*, CTNNB1* (*P* < 0.0001)*, FMN2* (*P* = 0.0003)*, TP53* (*P* = 0.0011) and *ZFX4* (*P* = 0.0054), showed significant differences between mutation and wild type group (Fig. 3a), with the area under the receiver operating characteristic curves (AUCs) from 0.71 to 0.89 in the external validation set. In addition, similar differences were observed on the slide-level, except for *ARID1A* (Fig. 3b). The per-slide AUCs after aggregation by average predicted probability and percentage of tiles with positive classification were listed in Table 4.

**Table 3:** The performances of our models for gene mutation prediction.

GENE	Accuracy	Precision	Recall	*F*1-score	MCC
*ARID1A*	0.925	0.833	0.769	0.800	0.755
*ASH1L*	0.896	0.778	0.583	0.667	0.615
*CSMD1*	0.910	0.714	0.556	0.625	0.581
*CTNNB1*	0.910	0.895	0.810	0.850	0.788
*EYS*	0.925	0.800	0.500	0.615	0.596
*FMN2*	0.925	0.727	0.800	0.762	0.719
*MDM4*	0.925	0.750	0.429	0.545	0.532
*RB1*	0.940	0.800	0.571	0.667	0.646
*TP53*	0.925	0.895	0.850	0.872	0.820
*ZFX4*	0.910	0.846	0.733	0.786	0.732

*MCC* Matthews correlation coefficient.

**Table 4:** The performance of our models at mutation prediction in the external validation set.

Mutations	Per-tile AUC	Per-slide AUC after aggregation by	
		Average predicted probability	Percentage of positive tiles
*CTNNB1*	0.805 (0.759–0.851)	0.898 (0.810–0.986)	0.817 (0.713–0.922)
*FMN2*	0.727 (0.666–0.789)	0.737 (0.613–0.861)	0.838 (0.742–0.935)
*TP53*	0.736 (0.696–0.777)	0.770 (0.650–0.890)	0.715 (0.591–0.840)
*ZFX4*	0.720 (0.675–0.765)	0.724 (0.591–0.858)	0.751 (0.614–0.888)

## Discussion

In this study, the deep-learning classifiers displayed a high-level performance at recognizing cancer apart from normal liver tissue and assessing histopathological grade (well, moderate, or poor). The performances nearly reached the ability of pathologists with 5-year experience. Interestingly, the model found 9 out of 13 WSIs from our center with grading misclassified by at least a pathologist. Although the sensitivity and accuracy still need to be improved to be on par with a 10-year experience pathologist, it could be used to assist young pathologists at diagnosing with shorter learning curve period, faster speed, and higher accuracy. Moreover, the prediction of the four genes mutation (*CTNNB1*, *FMN2*, *TP53*, and *ZFX4*) is beyond the ability of pathologists.

The prediction of mutation based on histopathological H&E images using deep learning may have a positive influence on the diagnosis and treatment of patients with cancer given the importance of gene mutation^21,27^. For example, the mutations in *CTNNB1* occurred at a relatively high frequency in HCC, with a high expression of the protein kinase human monopolar spindle 1 (*hMps1/TTK*), and *TTK* inhibitors regarded as one of the potential targeted drugs for *CTNNB1* mutant HCC^28–30^. Interestingly, our models showed a high-performance level of predicting *CTNNB1* mutation. The prediction of *CTNNB1* mutation using deep learning may make a great contribution to select patients who are most likely to respond to *TTK* inhibitor targeted therapy.

Due to the unclear AI algorithmic data processing in a “black box”, developers and users do not know how computers arrive at conclusion, thereby making it difficult to find out the detail of evidence resulting in a conclusion^31,32^. Therefore, as a novel tool for diagnosis and treatment, AI should be validated against current quality standards to ensure clinical effectiveness and safety in clinical practice^33,34^. In this study, an independent database from our center was used to validate the performance of our models. It was demonstrated that convolutional neural networks could be used to assist in the classification and mutation prediction, based on histopathological H&E slides in liver cancer. However, the model still needs to be improved and validated by larger studies in the future. Even though it is impossible for AI to completely replace humans in practice nowadays, it is still a useful and effective tool to assist clinicians in dealing with repetitive work to provide important prognostic and therapeutic information. For example, mutation prediction could serve as pre-screening to improve cost-efficiency before immunohistochemistry or next-generation sequencing.

Overall, the study demonstrates that convolutional neural networks can predict histopathological grade and mutation in liver cancer. Although AI is likely to be a useful tool to assist surgeons and pathologists in classification of WSIs of HCC, the black box that how to get the conclusion is unclear and should be further studied. Besides, it is the first study to predict the gene mutation in HCC, meanwhile, internal and external validation cohorts were utilized to improve the accuracy of the model. In addition, the information on pathology and gene mutations may potentially be significant in applying the appropriate targeted therapy to HCC patients, thereby improving the performance of precision medicine.

The present study has several limitations to discuss. On the one hand, the size of the validation cohort is small. On the other hand, the model is not a complete replacement for pathologists’ examination, which included the diversity and heterogeneity of tissues that pathologists typically inspect (e.g., inflammation, necrosis, and blood vessels) and some clinical factors. Therefore, further validation of our model is necessary in a larger dataset with multiple centers and clinical factors or characteristics should be considered in further study. Moreover, EASY DL platform is exclusively available in Chinese which considerably limits the scope and audience targeted. To address the limitation, we provided the step-by-step instruction (figures and detailed English descriptions) for training deep-learning models via EASY DL, which was available at GitHub (https://github.com/drmaxchen-gbc/HCC-deep-learning/) named “How_to_use_EASY DL”.

In conclusion, our study demonstrated that the convolutional neural networks could assist pathologists in the classification of liver cancer and the detection of gene mutation. It also revealed that this method might be successfully adopted for other types of solid tumors.

## Methods

### Prepare histopathological tiles dataset of liver cancer

**Fig. 4:**
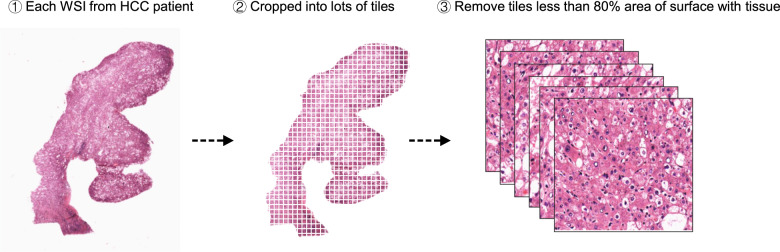
Strategy of preparing tiles dataset. First, each WSI of liver tissue was selected from GDC-portal or SRRSH. Then, they were cropped into lots of tiles. Finally, the tiles less than 80% area of surface with tissue were removed, and the remaining tiles were used for further analysis.

The frozen slide images and the corresponding cancer information were obtained from the GDC-portal (https://portal.gdc.cancer.gov/). On slide-level, there were 491 WSIs (HCC vs. normal liver tissue, 402 vs. 89), 383 WSIs of HCC with available histopathological grade (well vs. moderate vs. poor, 55 vs. 187 vs. 141) and 387 WSIs of HCC with corresponded gene mutation information. Besides, 67 WSIs of HCC with completed information and 34 WSIs of normal liver tissue were selected from Sir Run-Run Shaw Hospital. All WSIs should be cropped into multiple small “tiles” at a magnification of 20×. The majority of slides could be cropped into more than 200 “tiles” on region (tiles)-level (Supplementary Fig. 1). Each tile was saved as a JPG format by nonoverlapping 256 × 256-pixel windows. In order to avoid heterogeneity, each tile, where less than 80% of the surface was covered by tissue, should be removed (Fig. 4). Finally, the liver cancer tiles dataset consisted of four subsets, including the training, testing, internal validation, and external validation sets. The data in the training and internal validation cohorts from the Genomic Data Commons portal (https://portal.gdc.cancer.gov/) were publicly available without restriction, authentication or authorization. The independent external validation cohort we used consisted of slide images without identifiable information and all participants had provided written informed consent. Our study was approved by the SRRSH of Medicine Institutional Review Board (KY20181209-5).

### Technical detail on frozen slides in the external validation cohort

The obtained specimens (e.g., liver tissues) were macroscopically examined, measured, sectioned through their longest axis, and then midsections were examined. The material was frozen at −28 °C, cut into 5–10 µm thick sections, Hematoxylin-Eosin (H&E) stained, and then analysed by pathologists with the light microscope. There were 67 out of 70 patients diagnosed as HCC and the related frozen slide were collected. Notably, normal liver tissues cannot be available in half of the obtained specimens, because normal liver tissues should be at least 2 cm away from tumors. Therefore, there were only 34 WSIs of normal liver tissues. In order to obtain digital pathology images, each slide was scanned at a magnification of 20× by using digital pathology scanner VS120 (Olympus).

### Deep-learning with convolution neural networks

Typical convolutional neural networks contain several levels of convolution filters, pooling layers, and fully connected layers. In our study, we primarily used inception V3 architecture, which makes use of inception modules which are made from a spread of convolutions having different kernel sizes and a max-pooling layer. The initial five convolution nodes are combined with two max-pooling operations and followed by 11 stacks of inception modules. A fully connected layer to the end of the inception modules was then added to permit us to utilize the pre-trained model and finetune the parameters for our own task. Finally, a softmax layer was added as a classifier outputting a probability for every class, and the one with the highest probability was chosen as the predicted class.

We used the pre-trained model offered by TensorFlow and finetuned it using histopathological images. It was pre-trained on the ImageNet dataset and available at the TensorFlow-Slim image classification library (http://tensorflow.org). We initialized the parameters from the pre-trained model because pre-training can speed up the convergence of the network. Most importantly, it was difficult to train a deep network with a small number of images due to the massive number of network parameters.

### Comparison with pathologists

One hundred and one WSIs of liver tissues without a label from the external validation cohort were used to test pathologist’s performance and compared with our model performance. All pathologists should report whether there is HCC, and if there is HCC, they should report histopathological grade via digital pathology images. The outcomes reported by six pathologists with 2-years, 5-years, and 10-years experience (two pathologists in each category) and our model were collected and analyzed by the R 3.6.0 (https://www.r-project.org). Cohen’s Kappa analysis was performed to assess inter-observer agreement. Good inter-operator agreements were observed in pathologists with 2-year experience (Kappa = 0.894; 95% CI, 0.837–0.944), pathologists with 5-year experience (Kappa = 0.933; 95% CI, 0.888–0.975), and pathologists with 5-year experience (Kappa = 0.967; 95% CI, 0.930–0.992).

### Identification of significantly mutated genes

**Fig. 5:**
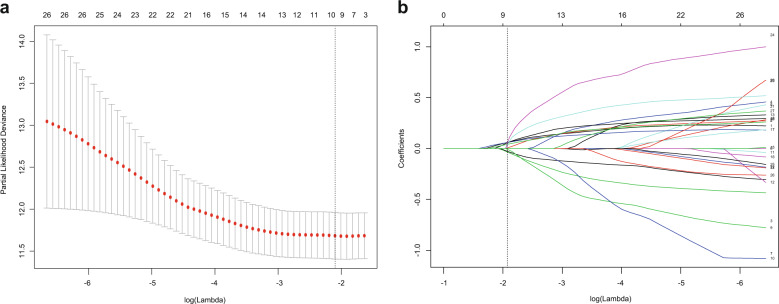
Prognosis-related mutated genes selection using the least absolute shrinkage and selection operator (LASSO) Cox regression model. **a** Selection of the super parameter *λ* in the LASSO model via 10-fold cross-validation based on the minimum standard. The optimal *λ* value of 0.122. **b** Shown here is a coefficient section view plotted against the log(*λ*) magnitude. The optimal *λ* corresponding to ten non-zero coefficients were obtained where the vertical line was drawn.

The gene mutation data for the matched patient sample were downloaded from the cancer genome atlas (TCGA). The gene mutated at least 10% of the available liver cancer samples were selected from the 283 cancer-related genes (Supplementary Fig. 2). The least absolute shrinkage and selection operator (LASSO) regression with a 10-fold cross-validation method was then performed to identify significant prognosis-related gene mutations by using R software packages (http://www.r-project.org). Finally, the ten most significant prognosis-related gene mutations, including *ARID1A, ASH1L, CSMD1, CTNNB1, EYS, FMN2, MDM4, RB1, TP53*, and *ZFX4* were identified (Fig. 5).

### Training deep-learning network

Pathological diagnosis was the primary endpoint of interest for the classifier that recognizes tumors from normal liver tissue and the assessment of the histopathological grade. The status of gene mutation (mutation or wild type), based on the next-generation sequencing results, was the primary prerequisite in the classifier of mutation prediction. The model’s training strategy was based on an easy-to-use platform called EASY DL (https://ai.baidu.com/easydl/) that uses PaddlePaddle deep learning framework V3.0 created by Baidu Brain AI technology, inception V3 network developed by Google, and packaging code form Coudray^20^ and co-workers. The training set was used for training, and the testing set was used to evaluate the performances, finetune those parameters, and improve the models. A final model was selected according to the results of the testing set, where the *F*1-scores as a stopping rule. Notably, the subsets were grouped based on HCC patients rather than the WSIs. This method could maximize the size of the training set and avoid training and testing on tiles originating from the same human subjects. Thereby preventing the classifier from relying on intra-subject correlations between samples and resulting in inflated estimates of accuracy. In order to reduce selection bias, the performance of our model was then validated in the internal and external validation sets.

### Statistical analysis

The ten most common and prognostic mutated genes were identified using the LASSO Cox regression model, and any differences of overall survival were evaluated by the Kaplan–Meier method with a log-rank test. The performance of those models was evaluated with *F*1-scores, MCC, and AUC. The *F*1-scores, ranging from 1 (perfect) to 0 (bad), is the harmonic average of the precision and recall^21^. MCC ranges from 1 (perfect) to −1 (bad). In addition, the probability of gene mutation was estimated and compared using the two-tailed Mann–Whitney *U*-tests. A *P* value of less than 0.05, was considered as statistical significance.

## Supplementary information

Supplementary Figures

## Data Availability

The slide images and the corresponding cancer information were uploaded from the Genomic Data Commons portal (https://portal.gdc.cancer.gov/) and were in whole or in part based upon data generated by the TCGA Research Network (http://cancergenome.nih.gov/). These data were publicly available without restriction, authentication, or authorization. The datasets for the independent cohorts generated and/or analyzed during the current study are available from the corresponding author (X.J.C.) upon reasonable request and through collaborative investigations.

## Code availability

The codes that were used to train and validate the deep-learning model in the manuscript are available at https://github.com/drmaxchen-gbc/HCC-deep-learning. It also used other open-source codes (inception V3), which were available at https://github.com/openslide/openslide-python.
