# Supplementary material for: Classification and mutation prediction based on histopathology H&E images in liver cancer using deep learning
Source: NPJ Precis Oncol. 2020 Jun 8;4:14. doi: 10.1038/s41698-020-0120-3 (PMC7280520; doi:10.1038/s41698-020-0120-3)
Supplement: Supplementary file 1 — Supplementary Figures [file 41698_2020_120_MOESM1_ESM.pdf]

Supplementary Figures Legends

Supplementary Figure 1. The distribution of the number of “tiles” per slide.

Supplementary Figure 2. 27 genes where mutated at least 10% of the available liver cancer.

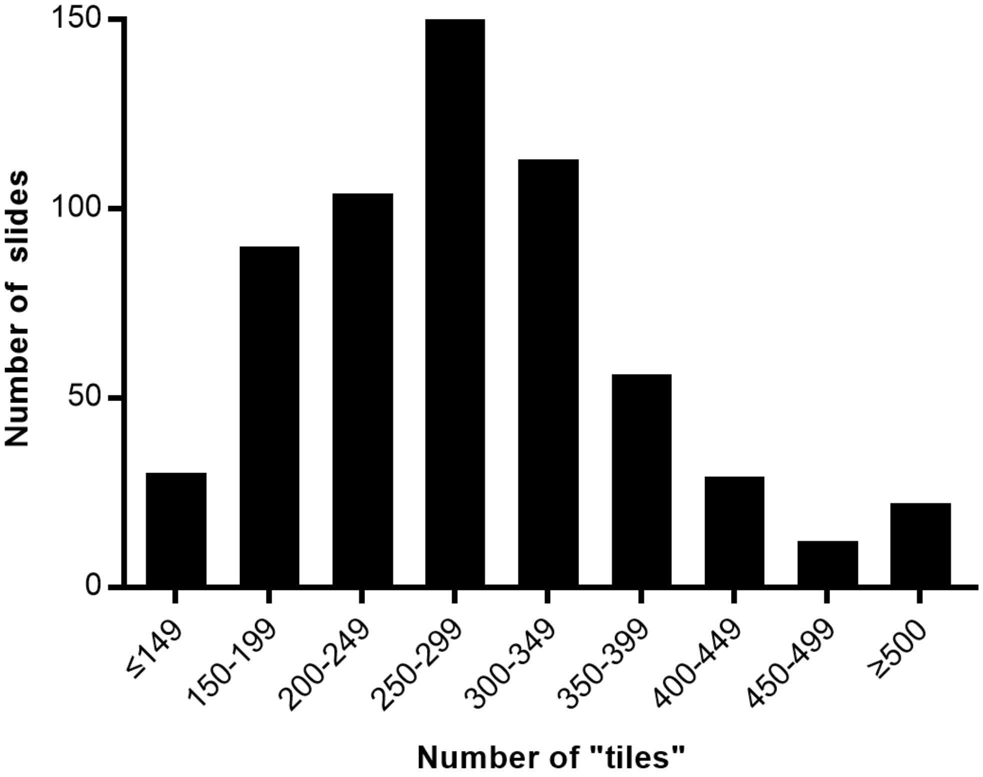

Supplementary Figure 1. The distribution of the number of “tiles” per slide.

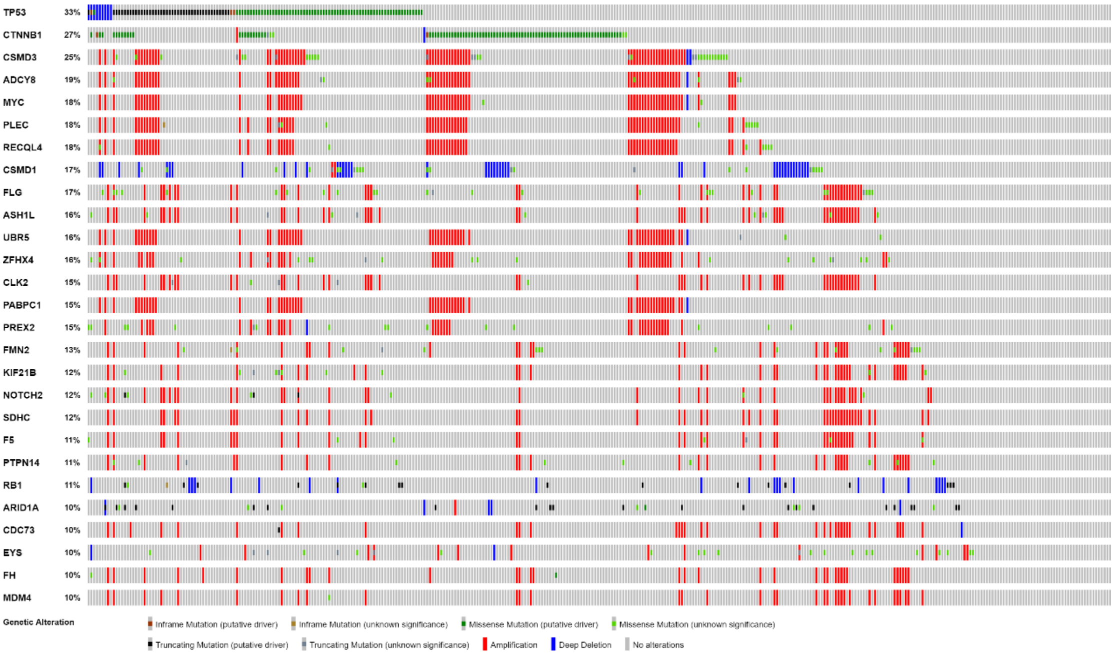

Supplementary Figure 2. 27 genes where mutated at least 10% of the available liver cancer.
